# Mechanisms of regulation of motility of the gastrointestinal tract and the hepatobiliary system under the chronic action of nanocolloids

**DOI:** 10.1038/s41598-023-30958-5

**Published:** 2023-03-07

**Authors:** Olga V. Tsymbalyuk, Tamara L. Davydovska, Anna M. Naumenko, Ivan S. Voiteshenko, Stanislav P. Veselsky, Alex Y. Nyporko, Anastasiia Y. Pidhaietska, Mariya S. Kozolup, Valeriy A. Skryshevsky

**Affiliations:** 1 Institute of High Technologies, Taras Shevchenko National University of Kyiv, 64, Volodymyrska Str., Kyiv, 01033 Ukraine; 2 Department of Foreign Languages for Sciences, Ivan Franko National University of Lviv, 41 Doroshenko St., Lviv, 79000 Ukraine; 3 Corporation Science Park, Taras Shevchenko University of Kyiv, 60, Volodymyrska Str., Kyiv, 01033 Ukraine

**Keywords:** Biochemistry, Gastroenterology, Nanoscience and technology

## Abstract

Modern cutting edge technologies of chemical synthesis enable the production of unique nanostructures with excess energy and high reactivity. Uncontrolled use of such materials in the food industry and pharmacology entail a risk for the development of a nanotoxicity crisis. Using the methods of tensometry, mechanokinetic analysis, biochemical methods, and bioinformatics, the current study showed that chronic (for six months) intragastrical burdening of rats with aqueous nanocolloids (AN) ZnO and TiO_2_ caused violations of the pacemaker-dependent mechanisms of regulation of spontaneous and neurotransmitter-induced contractions of the gastrointestinal tract (GIT) smooth muscles (SMs), and transformed the contraction efficiency indices (AU, in Alexandria units). Under the same conditions, the fundamental principle of distribution of physiologically relevant differences in the numeric values of the mechanokinetic parameters of spontaneous SM contractions between different parts of GIT is violated, which can potentially cause its pathological changes. Using molecular docking, typical bonds in the interfaces of the interaction of these nanomaterials with *myosin II*, a component of the contractile apparatus of smooth muscle cells (SMC) were investigated. In this connection, the study addressed the question of possible competitive relations between ZnO and TiO_2_ nanoparticles and actin molecules for binding sites on the *myosin II* actin-interaction interface. In addition, using biochemical methods, it was shown that chronic long-term exposure to nanocolloids causes changes in the primary active ion transport systems of cell plasma membranes, the activity of marker liver enzymes and disrupts the blood plasma lipid profile, which indicates the hepatotoxic effect of these nanocolloids.

## Introduction

Nanocrystal metal oxides, including TiO_2_, constitute an important class of inorganic substances, in view of the great diversity of structures based on them. Today, with the development of modern innovative technologies, TiO_2_ is increasingly used in various fields, including the food industry, pharmacology, medicine, and biotechnology. This synthetic material has such characteristic features as a significant specific surface, a well-developed system of channels, an ability to peptize in electrolyte solutions, readily hydrolyze, and form stable colloidal solutions with various amphoteric hydroxides, etc.^1–3^. In such structures, oxygen vacancies, internodal Ti^**4**+^ and Ti^**3+**^ ions are the platforms for the adsorption of predominantly hydrophobic amino acids of protein macromolecules, accompanied by the unfolding and modulation of their secondary structure, and the formation of nanocomposites—dynamic adsorption–desorption complexes with controlled morphological, physical and chemical properties^3–8^. The development of technologies for the delivery of organic molecules into cells in the modern pharmaceutical industry is based on the phenomenon of their adsorption on the surface of such material^9–14^. By adsorbing macromolecules of receptor proteins and forming bonds with the binding sites of their allosteric modulators, TiO_2_ NPs block the agonist's access to its binding site, thereby disabling the launch of intracellular signaling cascades—a vital link for the physiological functioning of a particular cell, tissue, or organ^9,10^. Our previous studies^2,15^ demonstrated that even under short-term intragastical burdening of rats with TiO_2_ AN, the mechanisms of motility regulation of the gastrointestinal tract become the targets for their action. Under such conditions, smooth muscles of the stomach and the large intestine are the primary sites of Ti^2+^ accumulation as reported in our previous study^15^. Besides nanocrystal TiO_2_, another commercially available synthetic material—nanocrystal ZnO—has unique physical and chemical properties, namely high adsorption capacity, considerable catalytic activity, chemical stability, and pH-dependent tunable surface charge^16,17^. Due to these features, its action (via food additives and medicines^18–22^) on GIT with different pH values in different parts can be unpredictable. With this in view, the purpose of our study was to address the following previously unresolved problems: (1) peculiarities of the development of pathological states of the mechanisms regulating the motility of the GIT and hepatobiliary system under chronic burdening with ZnO and TiO_2_ ANs, (2) the state of chemoreceptors, which are the target components of most medicines, under effect of these nanocolloids, and (3) the potential competitive relations between ZnO and TiO_2_ ANs and the molecules of the contractile apparatus, which may impair the mechanisms of regulation of the *myosin II-actin* interface formation with the formation of actomyosin.

## Results and discussion

### Study of the state of spontaneous contractile activity of the gastric and large intestine smooth muscles of rats under the chronic burdening with ZnO and TiO_2_ nanocolloids

**Figure 1:**
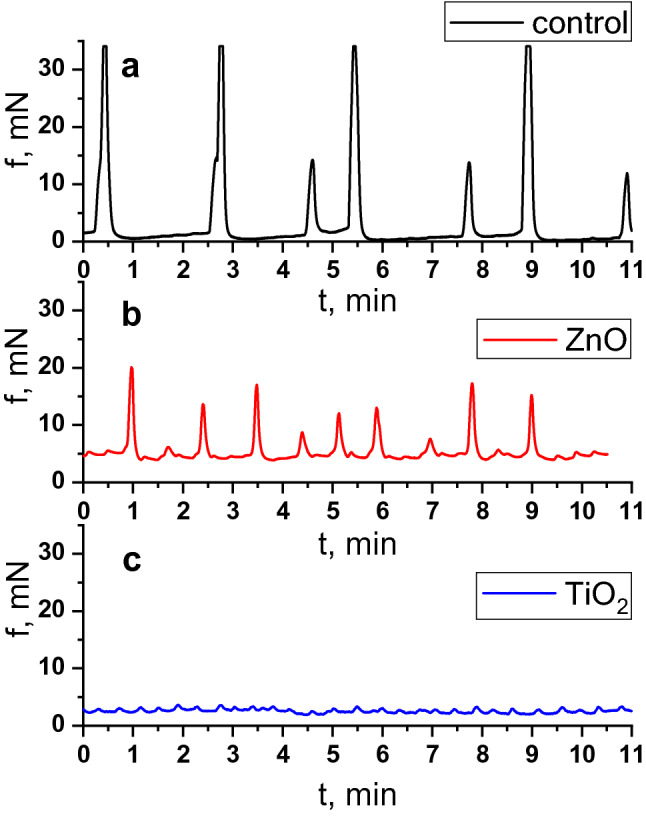
Spontaneous contractile activity of the circular SMs of the *antrum* of rats: (**a)** control group; (**b**) and (**c**) groups of rats burdened for 6 months with ZnO and TiO_2_ ANs, respectively.

**Figure 2:**
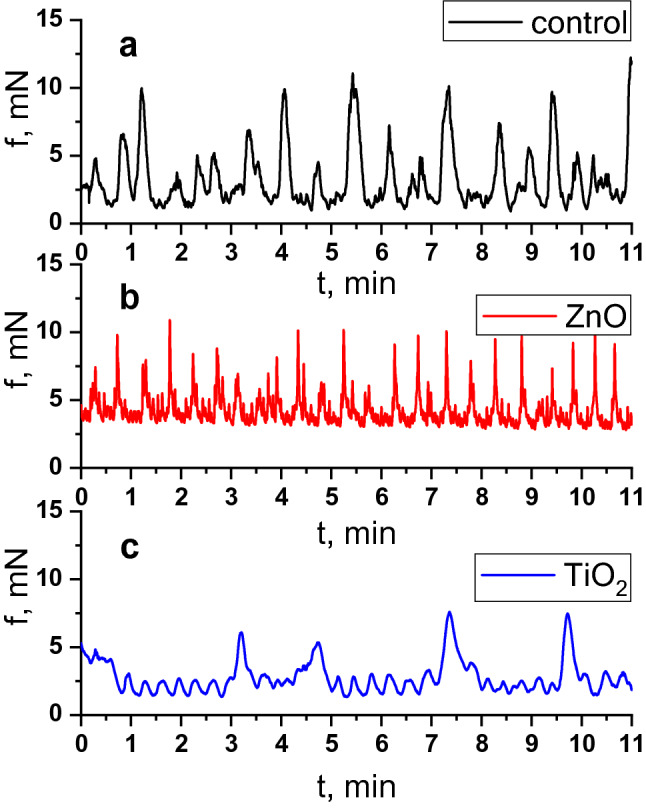
Spontaneous contractile activity of the circular SMs of the *caecum* of rats: (**a**) control group; (**b**) and (**c**) groups of rats burdened for 6 months with ZnO and TiO_2_ ANs, respectively.

**Figure 3:**
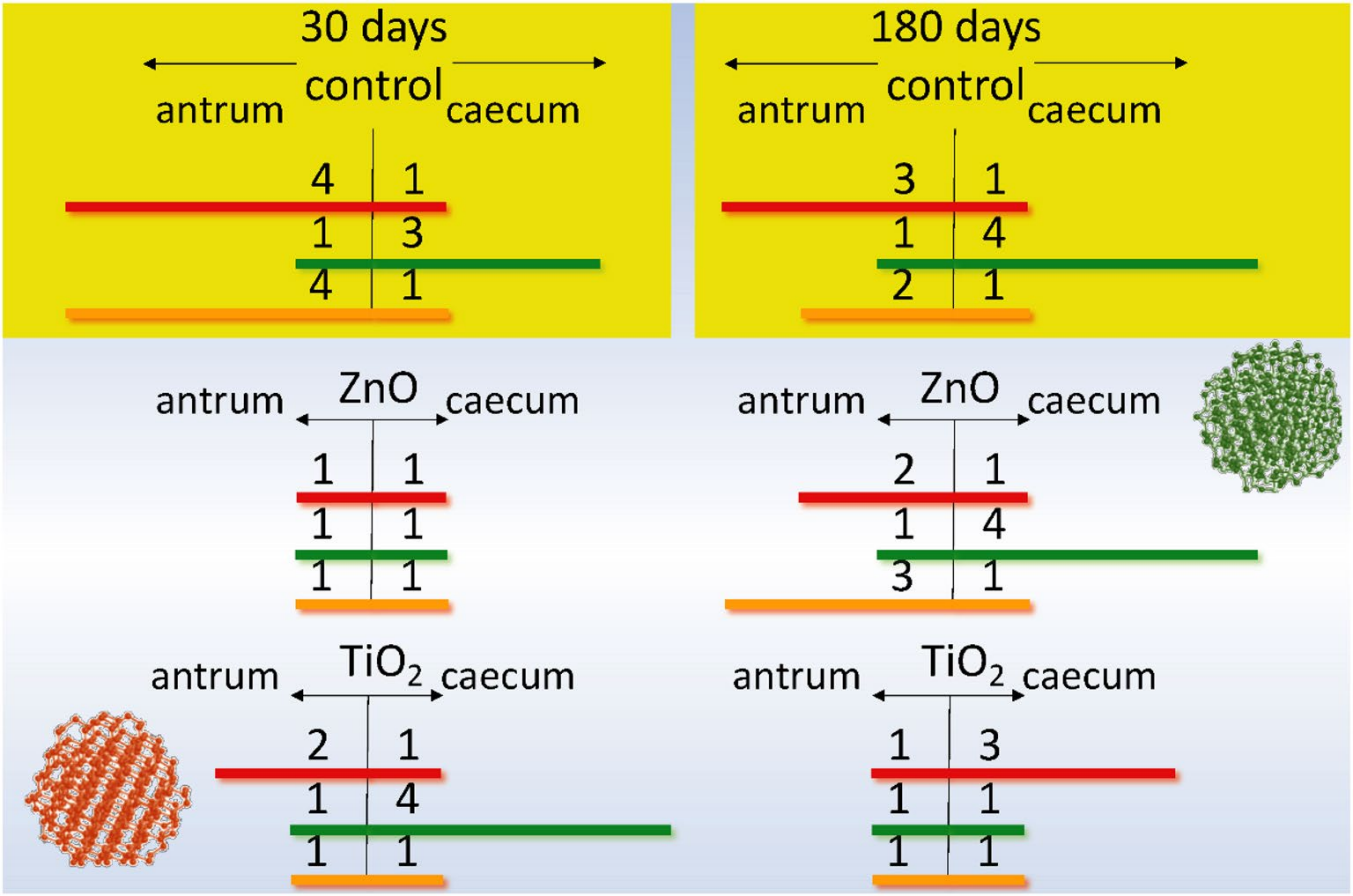
Schematic representation of the ratio of the parameters of spontaneous SM contractions of the *antrum* and *cecum* of rats in the control group and in the group intragastrically burdened with TiO_2_ and ZnO ANs for six months: **red**—AU contraction indices; **green**—the frequency of spontaneous contractions of preparations per 10 min.; **orange**—an averaged value of the duration of contraction-relaxation cycle. A comparison of these and our previous study^2^ results whereby rats were intragastrically burdened with ANs for 30 days.

The generation of spontaneous electric and contractile activity of the gastrointestinal tract SMs involves interstitial cells of Cajal (ICC)—components of a heterogeneous population of cells of GIT wall—and is regulated by the vegetative and enteric nervous systems^23–25^. Various types of ICC form populations with a clearly defined spatial location in different parts of GIT^25–27^. Pacemaker activity of such cells is associated with oscillations of the intracellular concentration of Ca^2+^ ions, with the involvement of nonselective cation TRPC4 channels located near caveolae^28,29^. In this study, spontaneous contractions of circular SMs of the gastric *antrum* and *caecum* of rats were registered using the tenzometric method in the isometric mode. It was found that the ratios of mechanokinetic parameters of spontaneous contractions of the *antrum pyloricum* SMs (ISS-DMP) (Fig. 1a) and those of the *caecum* (ISS-SMY) (Fig. 2a) differ significantly (Fig. 3). Thus, the ratio of the averaged values of frequency of spontaneous contractions of the *antrum piloricum* and *caecum* SMs was 1:4, *n* = 12 (Figs. 4.1, 5.1), the ratio of durations of contraction-relaxation cycles—2:1, *n* = 12 (Figs. 4.2, 5.2). The amplitude ranges were within (25–34) mN, *n* = 12, and (0.7–10) mN, *n* = 12, respectively. The averaged values of the amplitudes of spontaneous contractions of the *antrum piloricum* SMs were significantly higher than those of the *caecum* SMs: (29.8 ± 1.12) mN, *n* = 12 and (4.4 ± 0.38) mN, *n* = 12 (Figs. 1a, 2a), respectively, which is consistent with the data in the literature^28–30^. The ratio of these parameters was 7:1. The averaged values of AU indices also differed (Fig. 3). The value of this parameter was the greatest for the *antrum piloricum* SMs (6217.8 ± 452.3), *n* = 12 (Fig. 4.3), while for *caecum* SMs it was (2458.7 ± 173.9), *n* = 12 (Fig. 5.3). The above results indicate the distribution of physiologically relevant differences in numeric values of mechanokinetic parameters and AU indices for SMs in different parts of GIT, which is vital for the formation of pressure gradients in them for the movement of chyme residues, as well as the filtration of solutions from the gastric cavity and intestines to blood and lymph.

**Figure 4:**
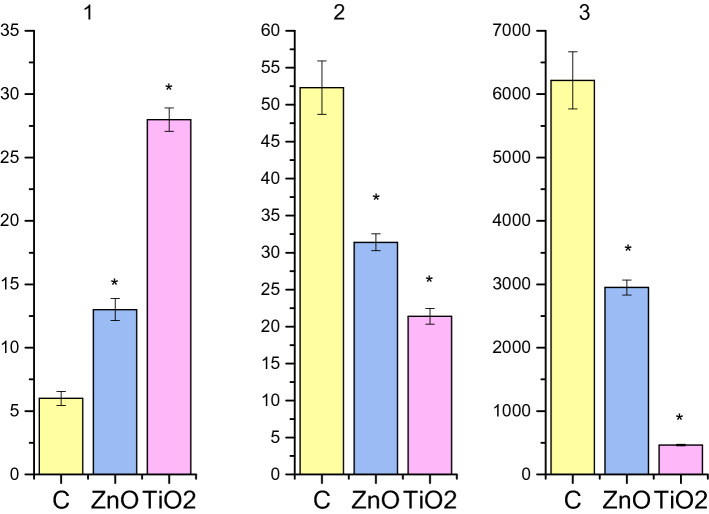
Histograms of the kinetic parameters for the spontaneous contractile activity of the circular SMs of the *antrum* of rats burdened with ZnO and TiO_2_ ANs for 6 months: C—control; (**1**) the frequency of contractions of preparations per 10 min.; (**2**) an averaged value of the duration of the contraction–relaxation cycle; (**3**) AU index of contractions; *p < 0.05 indicates reliability of changes as compared with the control.

**Figure 5:**
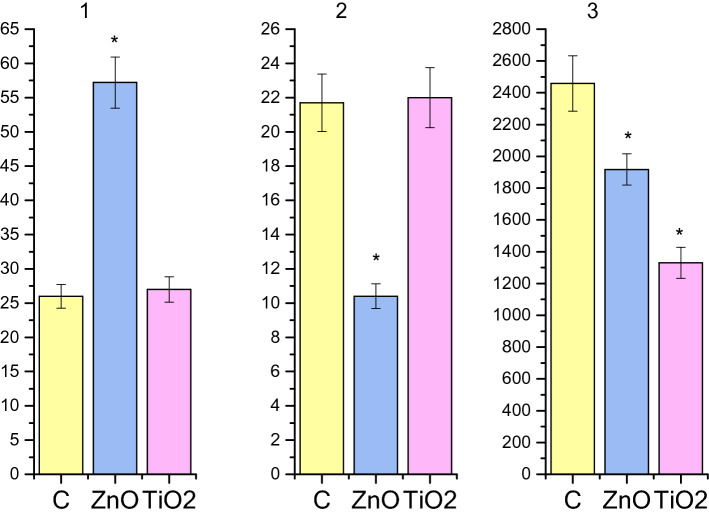
Histograms of the kinetic parameters for the spontaneous contractile activity of the circular SMs of the *caecum* of rats burdened with ZnO and TiO_2_ ANs for 6 months: C—control; (**1**) the frequency of contractions of preparations per 10 min.; (**2**) an averaged value of the duration of the contraction–relaxation cycle; (**3**) AU index of contractions; *p < 0.05 indicates reliability of changes as compared with the control.

According to the literature^31^, age-related changes in the body cause partial loss of ICC (13% per decade for the human body aged 25–74 years) and the development of pathologies. A comparison of the above values of the spontaneous contractions parameters of the *antrum piloricum* and *caecum* SMs of rats (control group, age eight months) with the values of the corresponding parameters in rats of the younger age group (control group, age three months), reported in our previous study^2^, showed that in the older age group of rats, the frequency of spontaneous contractions of both the *antrum pyloricum* and the *caecum* SMs was almost twice lower than in the younger age group, *n* = 12. The duration of contraction-relaxation cycles increased; the amplitude ranges were nearly identical, whereas the amplitudes of contractions of the *antrum* SMs were three times higher than the corresponding value for the younger age group. In the studied parts of GIT, the ratio of AU contraction indices in the older and younger age groups was different (Fig. 3).

**Figure 6:**
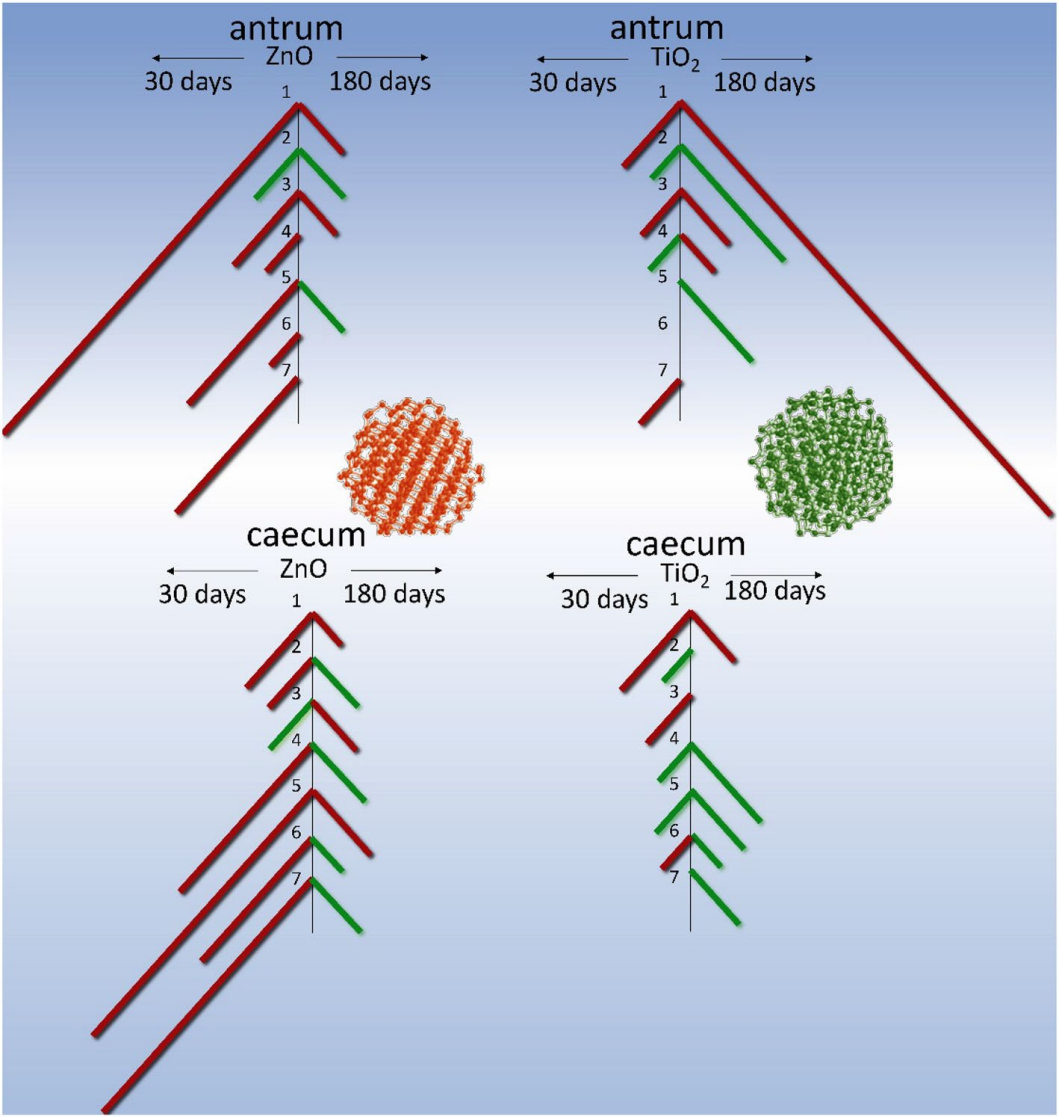
Scheme comparing the changes in the parameters of spontaneous and induced by acetylcholine (10^–5^ M) and high-potassium Krebs solution (80 M) contractions of circular smooth muscles (SM) of the antrum and cecum of rats burdened by ZnO and TiO_2_ ANs for for 6 months: (**1**) changes in AU contraction indices, (**2**) frequency of spontaneous contractions, (**3**) duration of the contraction-relaxation cycle; (**4, 5**) changes in the phase and tonic components of acetylcholine-induced SM contractions; (**6, 7**) changes in the phase and tonic components of high-potassium contracture of SMs. The inhibition and enhancement of the above parameters compared to control are highlighted in red and green, respectively. The figure also presents a comparison of changes in corresponding parameters of smooth muscles under burdening of rats with nanocolloids for 30 days (according to our previous study^2^).

In the next series of experiments, we investigated the state of mechanokinetic parameters of spontaneous contractions of the gastric *antrum* and *caecum* SMs of rats (older age group) intragastrically burdened with ZnO AN in doze 3 mg/kg/day of dry matter for six months. Under these conditions, there was a more than twofold increase in the frequency of spontaneous contractions of both the *antrum piloricum* (Figs. 1b, 4.1) and the *caecum* (Figs. 2b, 5.1) SMs compared to the control. The ratio of these parameters of spontaneous contractions of different GIT parts remained within the control (Fig. 3). Compared to the control, the duration of the contractile cycle of both the *antrum piloricum* (Fig. 4.2) and the *cecum* (Fig. 5.2) SMs decreased by 2 times, *n* = 12, their ratio, which was 3:1, changed, *n* = 12 (Fig. 3). Under the same conditions, there was an almost fourfold decrease, *n* = 12 of the averaged value of the amplitude of spontaneous contractions of the *antrum piloricum* SMs compared to the control, while this parameter of the *caecum* decreased only by (32.3 ± 1.59 ) %, *n* = 12, *p* < 0.05. Their ratio was 1:1, while in the control it was 7:1. Compared to the control, the AU indices of the *antrum piloricum* (Fig. 4.3) and *caecum* SMs decreased and acquired such values, respectively: (2949.4 ± 116.7), *n* = 12, *p* < 0.05 and (1917, 3 ± 98.2), *n* = 12, *p* > 0.05 (Fig. 5.3). A comparison of the above results with our previously obtained data ^2^ about the state of the GIT motility mechanisms of the younger group of rats, which were intragastrically burdened with ZnO AN for 30 days, showed (Figs. 3, 6) that under the same conditions, in contrast to the older age group of animals, the younger one demonstrated significantly greater violations of the mechanisms regulating the maintenance of different numeric values of the mechanokinetic parameters of spontaneous contractions of the *antrum piloricum* and *caesum* SMs.

In the next series of experiments, the state of mechanokinetic parameters of spontaneous contractions of the *antrum piloricum* and *caecum* SMs of rats intragastrically burdened with TiO_2_ AN for six months was studied. We found (Figs. 1c, 4.1) that against the background of a significant decrease in amplitude (4.7 times, *n* = 12) compared to the control, the *frequency* of spontaneous contractions of the *antrum piloricum* SMs increased, while in the *caecum* the value of this parameter remained within control (Fig. 2c, 5.1). These parameters reached the values of the same order, and their ratio was 1:1, while in the control it was 1:4 (Fig. 3). Under the same conditions, the averaged value of the duration of the contraction-relaxation cycle of the *antrum* SMs decreased almost 2.5 times, *n* = 12; the ratio of these parameters for the *antrum pyloricum* and *caecum* also reached the values of the same order—1:1, while in the control it was 2:1 (Figs. 4.2, 5.2). AU contraction index of the *antrum pyloricum* SMs changed significantly—compared to the control, this parameter decreased by 16 times, *n* = 12, while AU index of the caecum SMs decreased by 2 times, *n* = 12. The ratio of the averaged values of these parameters changed as well – it was 1:3, while in the control it was 3:1 (Figs. 3, 4.3, 5.3). A comparison of the above results with the previously obtained data^2^ about the state of the mechanisms of regulation of the spontaneous contractile activity of SMs in different parts of GIT under short-term (30 days) intragastrically burdening of rats with TiO_2_ AN showed (Figs. 3, 6) that they underwent the greatest changes under the long-term exposure, while under otherwise the same conditions, the results of burdening with ZnO AN were the opposite. The general principle of the action of these nanocolloids consists in the modulation or complete leveling of physiologically relevant differences in numeric values for most mechanokinetic parameters of spontaneous contractions of the stomach and large intestine SMs, which can initiate the development of pathological states.

### Study of the state of acetylcholine-induced contractions of the smooth muscles of the stomach and large intestine of rats under the chronic action of ZnO and TiO_2_ nanocolloids

The M2, M3 receptor-dependent mechanisms of cholinergic excitation of smooth muscles also play an important role in the control and modulation of GIT motility^32,33^. Although the intestine is able to function even in the absence of external nervous influences^33,34^, the functioning of the stomach largely depends not only on the pacemaker activity of interstitial cells, but also on sympathetic and parasympathetic nerve pathways.With this in view, we investigated the effect of ZnO and TiO_2_ ANs on acetylcholine-induced (10^−5^ M) contraction-relaxation of the *antrum piloricum* and *caecum* SMs of rats intragastically burdened with these nanocolloids for six months.

**Figure 7:**
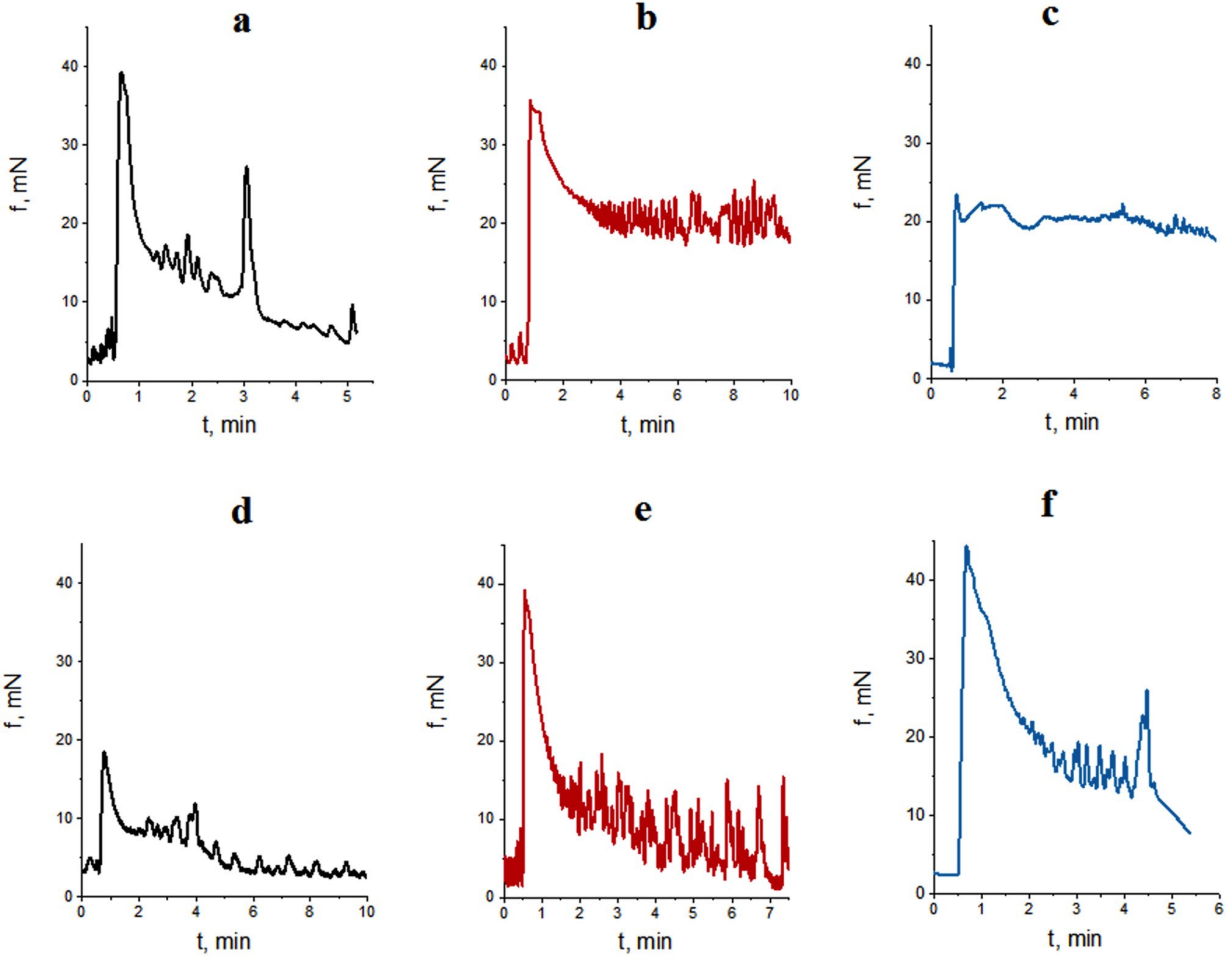
Acetylcholine-induced (10^−5^ M) contractions of SMs of the *antrum* and *cecum* of rats in the control group (**a, d)**, and in the groups intragastrically burdened with ZnO (**b, e**) and TiO2 (**c, f**) nanocolloids.

In the control (Fig. 7a), the averaged value of the phase component of contraction of the *antrum piloricum* SMs induced by acetylcholine (AC) was (31 ± 2.6) mN, *n* = 12, and that of the tonic component—(9.2 ± 0.7) mN, *n* = 12; whereas for the *caecum* SMs the values of these parameters were (13.8 ± 1.2) mN, *n* = 12 and (7.3 ± 0.5) mN, *n* = 12. The calculated normalized maximum velocities of contraction (Vnc)–relaxation (Vnr) were, respectively: (16 ± 1.38) min^−1^, *n* = 12, (3.96 ± 0.42) min^−1^, *n* = 12, and (10.4 ± 0.61) min^−1^, *n* = 12, (1.9 ± 0.1) min^−1^, *n* = 12. It was found (Fig. 7b) that under intragastric burdening of rats with ZnO AN there were no changes in the phase component and Vnc of acetylcholine-induced contractions of the *antrum piloricum* SMs, while the tonic component decreased almost 2 times, *n* = 12, and Vnr decreased by 5 times, *n* = 12, as compared to control. In the case of the *caecum* SM contractions, the phase component increased by 2.4 times, *n* = 12, and the tonic one increased by 2.6 times, *n* = 12 (Fig. 7e), compared to control (Fig. 7d). Under these conditions, compared to the control, Vnc increased by 1.7 times, *n* = 12, while Vnr decreased by 1.5 times, *n* = 12. In contrast to the action of ZnO AN, under intragastical burdening of rats with TiO_2_ AN, the phase component of acetylcholine-induced contraction of the *antrum piloricum* SMs decreased by more than 1.5 times, *n* = 12 in comparison with the control (Fig. 7a), but the speed of its growth increased, while the development of the tonic component was blocked (the curve reached the plateau) (Fig. 7c). Under the same conditions, the phase component of the *caecum* SM contractions increased by 3.2 times, *n* = 12, Vnc remained within control, while Vnr decreased 1.8-fold, *n* = 12 (Fig. 7f). The comparative analysis showed (Fig. 6) that in the younger age group there were significantly greater changes in the magnitude (direction) of the above parameters of acetylcholine-induced contractions of the *antrum* and *caecum* SMs under intragastical burdening of rats of the older age group with ZnO AN, whereas under the effect of TiO_2_ AN, these changes were of the same order (or were absent).

The recorded changes in the phase component of acetylcholine-induced contractions of SMs under the action of nanocolloids, considering the literature data^35,36^, can also be caused by changes in the conductance of voltage-gated Ca^2+^ channels (VGCs) to these cations. Indeed, in our experiments, we observed changes in the amplitude of high-potassium contracture of the *caecum* SMs (under the effect of ZnO) and both the *caecum* and *antrum piloricum* SMs (under the effect of TiO_2_) (Fig. 6). A probable reason for the changes in the tonic component of AC-induced contraction, can the action of nanocolloids on M2 receptor-dependent mechanisms limiting the flow of Ca^2+^ ions into the cytoplasm of cells via VGCs during cholinergic excitation^29^.

Another plausible reason for the changes in the phase component of AC-induced SM contraction under the action of ZnO and TiO_2_ ANs might be the impairment of the mechanisms activating the system of kinase/phosphatase in myosin light chains^35^, which are responsible for the activation of SM contractions. Considering the above, molecular docking of ZnO and TiO_2_ ANs with *myosin II* molecules was performed.

### Molecular docking of nanostructured ZnO and TiO_2_ with myosin II

**Figure 8:**
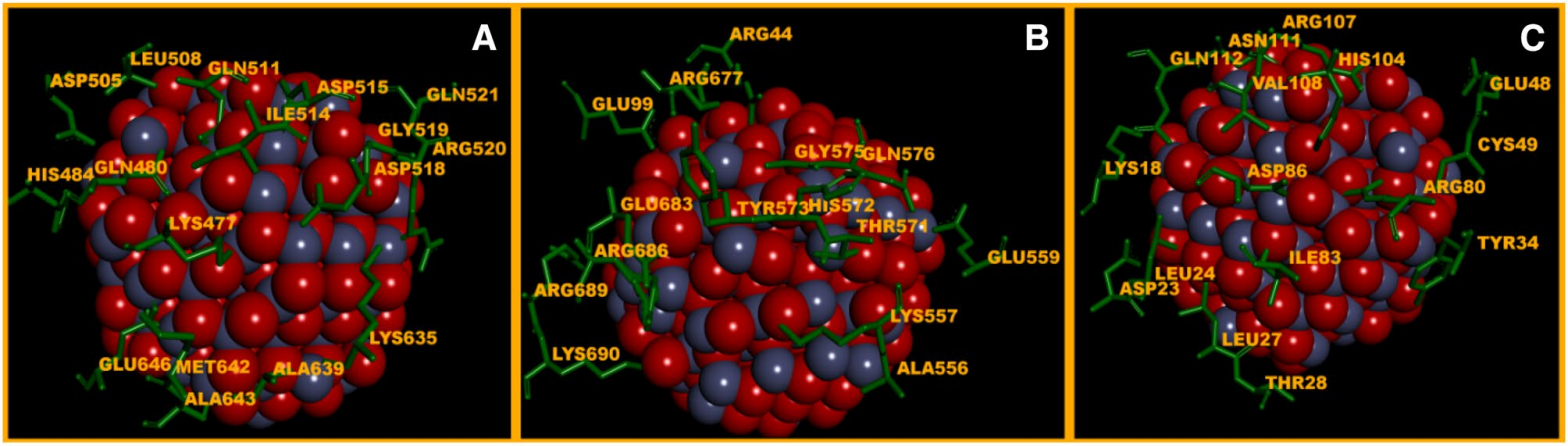
(**A**) The first binding site of ZnO with *myosin II* (Score—7982, Area—937.10 Å^2^, ACE–89.79); (**B**) the second binding site of ZnO with *myosin II* (Score—7524, Area—1013.40 Å^2^, ACE—159.34); (**C**) the third binding site of ZnO with *myosin II* (Score—7414, Area—1109.80 Å^2^, ACE—182.43).

Spherical ZnO and TiO_2_ NPs of 5 nm were simulated to identify the set of typical bonds: crystalline structure of the nanoparticle—*myosin II* Dictyostelium discoideum. Firstly, the selected form of the NPs ensured comprehensive investigation of coordination bonds of docking proper, and secondly, it allowed for less complicated and time-consuming calculations. In our work, three complexes of spherical ZnO NPs with *myosin II* were obtained by means of molecular docking. The first binding site and its environment included 17 amino acid residues (a.r.) (Fig. 8A), where the ratio of polar uncharged, non-polar, and polar charged a.r. was as follows: 0.18:0.35:0.47, and the respective distribution of the Gibbs free energy (kJ/mol) was: (− 0.99); (− 2.89); (− 2.76). The values of Geometric shape complementarity score (Score), Approximate interface area (Area) and Atomic Contact energy (ACE), calculated using PatchDock web-server, were as follows: 7982; 937.10 Å^2^ and 89.79, respectively. The second binding site of ZnO NPs with *myosin II* and its environment (Fig. 8B) also included 17 a.r.; their ratio in terms of polarity being: 0.18:0.12:0.7 and the distribution of the Gibbs free energy (kJ/mol): (− 0.57); (− 0.77); (− 5.22). In this case, the PatchDock web-server calculations were as follows: 7524; 1013.40 Å^2^ and 159.3. The third binding site of ZnO NPs with *myosin II* (Fig. 8C) included 16 a.r. with the ratio in terms of polarity: 0.31:0.25:0.44, and the distribution of the Gibbs free energy (kJ/mol): (− 0.91); (− 1.61); (− 2.64). The Score, Area and ACE values were as follows: 7414; 1109.80 Å^2^ and 182.43.

Our study also addressed the issue of the possible competitive relations between ZnO NPs and actin molecules for binding sites on the actin-binding site of *myosin II*. A comparative analysis based on our results and the data^37–39^ showed that ZnO NPs do not compete with actin for interaction interface in any of the three binding sites mentioned above. However, it was found that ZnO NPs can form bonds with amino acids in loop 519–524 of *myosin II*, namely: Gly519, Arg520, Gln521 (Fig. 8A). According to the data^39^, loop 519–524 is not the main binding site of actin with *myosin II*, but is a determining factor in its conformational transformations.

**Figure 9:**
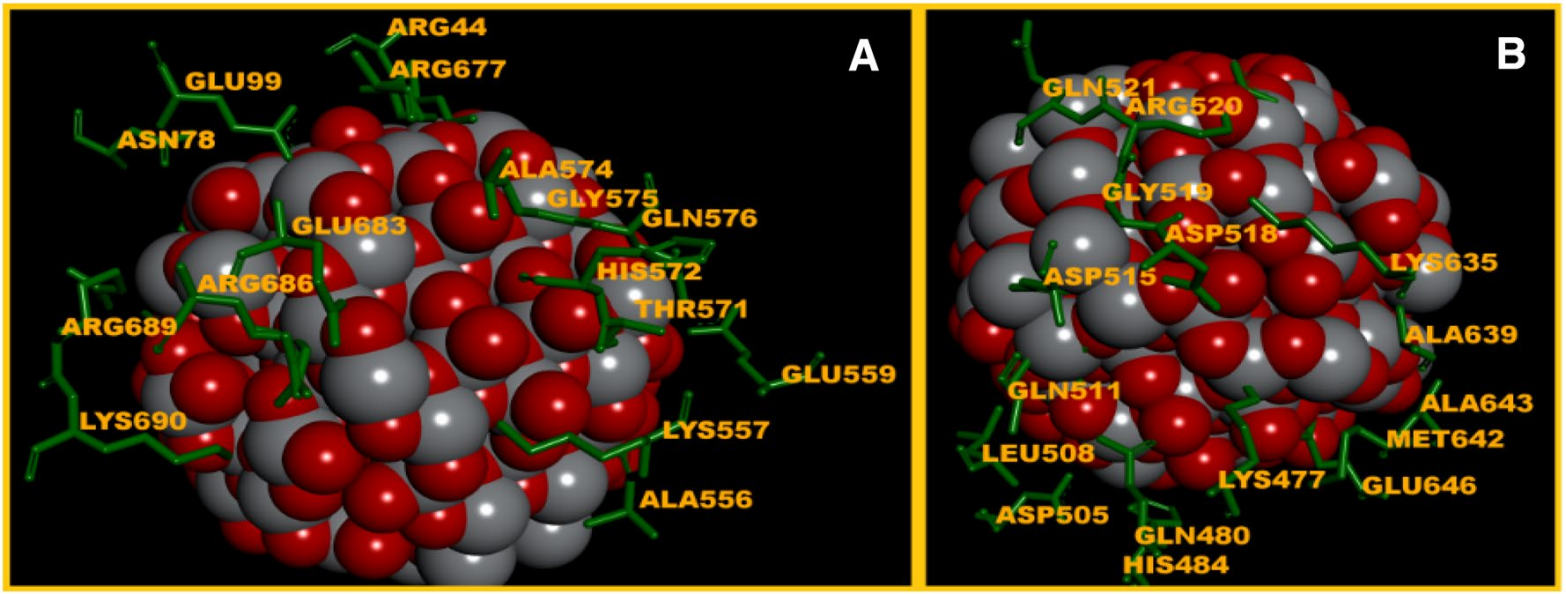
(**A**) The first binding site of TiO_2_ with *myosin II* (Score—7910, Area—1101.40 Å^2^, ACE—204.62); (**B**) the second binding site of TiO_2_ with *myosin II* (Score—7724, Area—947.20 Å^2^, ACE—246.42).

We obtained two complexes of spherical TiO_2_ NPs with *myosin II*. The conducted molecular docking showed that the first binding site included 19 a.r. (Fig. 9A); where the ratio of polar uncharged, non-polar and polar charged a.r. was: 0.16:0.16:0.68, and the respective distribution of the Gibbs free energy (kJ/mol) was: (− 0.51); (− 1.54); (− 5.84). The PatchDock calculations, namely Score, Area, and ACE, had the following values: 7910; 1101.40 Å^2^ and 204.62.

The second binding site of TiO_2_ NPs with *myosin II* and its environment (Fig. 9B) included 17 a.r.; their ratio in terms of polarity being: 0.19:0.31:0.5 and the distribution of the Gibbs free energy (kJ/mol): (− 0.99); (− 2.66); (− 2.76). The PatchDock calculations had the following values, respectively: 7724; 947.20 Å^2^ and 246.42. As in the case of ZnO NPs, a comparative analysis showed no competitive relations between TiO_2_ NPs and actin for binding sites on the actin-interaction interface of *myosin II*. However, it was found that both TiO_2_ and ZnO NPs, can form bonds with amino acids in loop 519–524 of *myosin II*, namely: Gly519, Arg520, Gln521 (Fig. 9B), which, as stated above, can cause the impairment of the mechanisms that regulate of the formation of the *myosin II-actin* interface.

### Study of biochemical markers for the liver function of rats under chronic exposure to ZnO and TiO_2_ nanocolloids

**Figure 10:**
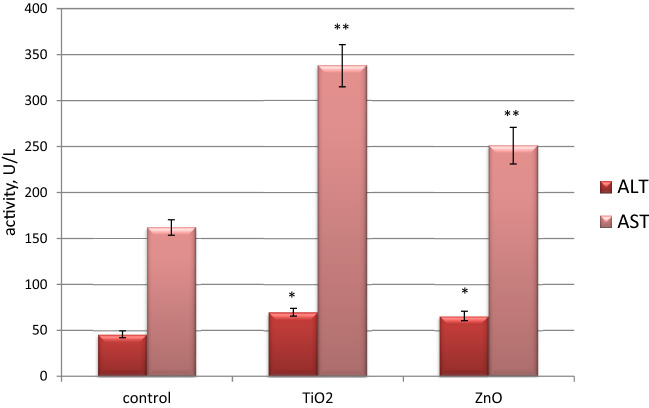
Activity of alanine aminotransferase (ALT) and aspartate aminotransferase (AST) in the blood plasma of rats in the control group (n = 7) and under the chronic in vivo effect of ZnO and TiO_2_ ANs for 6 months (n = 7); *p < 0.05 and **p < 0.001 compared with control.

Alanine aminotransferase (ALT) and aspartate aminotransferase (AST), which play a key role in the metabolism of virtually all amino acids and gluconeogenesis, are classical markers of the liver function. An increased activity of these enzymes in blood plasma is associated with the destruction of liver cells under the action of various toxic factors (xenobiotics, alcohol abuse, hepatitis of various genesis, impaired bile flow, etc.), besides, it is a predictor of the metabolic syndrome development against the background of impaired lipid metabolism^40,41^. Therefore, we investigated the activity of ALT and AST in the blood plasma of rats intragastically burdened with ZnO and TiO_2_ ANs. The activity level of ALT and AST enzymes of the control animals was 45.7 ± 4.1 U/l and 161.9 ± 8.3 U/l (n = 7), respectively. In both groups of experimental animals, a significant increase in the activity of these enzymes was observed (Fig. 10), and this effect was more pronouced under chronic exposure to TiO_2_ AN, which indicates a relatively higher hepatotoxic effect of this nanocolloid. It is noteworthy that in our previous studies^2^, a significantly greater increase in ALT and AST activity levels (4–6-fold and 2–2.5-fold, respectively) was detected in the blood plasma of rats intragastically burdened with ZnO and TiO_2_ ANs for 30 days. Thus, it can be assumed that hepatotoxic effect of these ANs will decrease over a longer period of exposure. Changes in the de Ritis ratio (the ratio of AST/ALT activities) confirm the regularity of the decrease of ANs’ hepatotoxic effects on the body of rats with the increase in the duration of their intake: under ZnO effect it was within the control range, while under TiO_2_ effect it was significantly increased.

### Lipid profile of blood plasma of rats under chronic exposure to ZnO and TiO_2_ nanocolloids

Another indicator of liver function impairment, along with ALT and AST activity indices, is the level of the main fractions of lipids in the blood—the features of intermediate lipid metabolism and lipid homeostasis. Therefore, we studied the composition of lipid fractions, namely phospholipids (PL), cholesterol (CHOL), higher fatty acids (HFA), triglycerides (TG) and cholesterol ethers (ECHOL), in the blood plasma of animals in the control and those exposed to ZnO and TiO_2_ ANs for 6 months.

**Figure 11:**
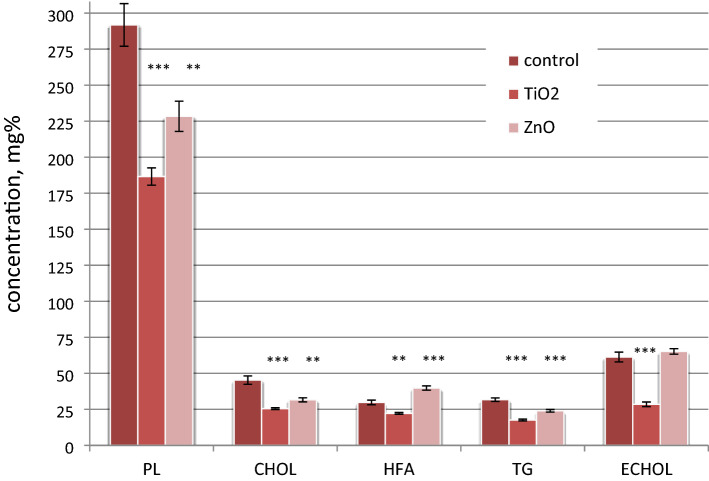
Concentrations of lipids (phospholipids (PL), cholesterol (CHOL), higher fatty acids (HFA), triglycerides (TG) and cholesterol ethers (ECHOL) in the blood plasma of rats in the control group (n = 7) and under the chronic in vivo effect of ZnO and TiO_2_ ANs for 6 months (n = 7); *p < 0.05 and **p < 0.001 compared with control.

Under chronic in vivo action of the ANs, the concentrations of total lipids decreased (under the effect of ZnO and TiO_2_—by 15.4% and 39%, respectively, *p* < 0.05, n = 7). TiO_2_ ANs caused a decrease in CHOL and ECHOL concentrations, while ZnO—only in CHOL (Fig. 11). These changes in the concentrations of CHOL and ECHOL impacted the controversial effects of the nanocolloids on the CHOL/ECHOL ratio, which decreased to 65.5% under the effect of ZnO (*p* < 0.001, *n* = 7), and increased to 120.7% under the effect of TiO_2_ (*p* < 0.05, *n* = 7).

Under the effect of the ANs, multidirectional changes in HFA concentration were observed: under the action of ZnO, the HFA level increased to 133.8% (*p* < 0.001, n = 7), while under the action of TiO_2_, it decreased to 74.6% (*p* < 0.01, n = 7). The chronic exposure to nanocolloids was also accompanied by a decrease in the PL level: under the action of ZnO—up to 78.3%, *p* < 0.01, n = 7), while under the action of TiO_2_—up to 63.9%, *p* < 0.001, n = 7). Both ANs caused a decrease in the level of triglycerides—the feature of oxidative stress activation, inflammation, and apoptosis of hepatocytes^42–44^. Thus, under the chronic in vivo action of ZnO and TiO_2_ ANs, lipid metabolism is inhibited, which indicates disorder in the liver function. In the case of transaminase activity, hepatotoxic effects of ZnO AN are significantly less pronounced.

### The activity of ATPases of the rat erythrocyte plasma membranes under the chronic effect of nanocolloids

**Figure 12:**
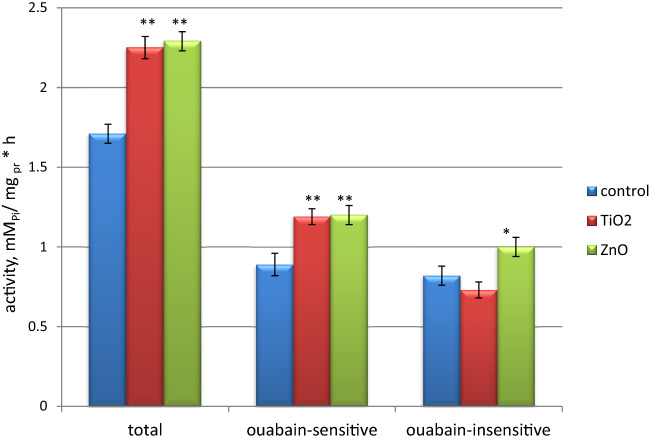
The total Mg^2+^-dependent, ouabain-sensitive (Na^+^,К^+^-ATPase) and ouabain-insensitive ATPase activity of erythrocyte plasme membranes of rats in the control group (n = 7) and under the chronic in vivo effect of ZnO and TiO_2_ ANs for 6 months (n = 7); *p < 0.05 and **p < 0,001 compared with control.

Systems of primary active transport of plasma membrane ions (Na^+^,К^+^-ATPase and Ca^2+^-pump) are critically important for functioning of cells as they provide transportation of glucose and amino acids, maintenance of the transmembrane gradient of ion concentrations, and hence the membrane potential^45^. In the control, the indices of total Mg^2+^-dependent, ouabain-sensitive and ouabain-insensitive ATPase activity were: (1.71 ± 0.06) mM P_i_/mg of protein·h, (0.89 ± 0.07) mM P_i_/mg of protein·h, and (0.82 ± 0.06) mM P_i_/mg of protein·h, respectively. Under the chronic action of ZnO and TiO_2_ ANs, an increase in the total Mg^2+^-dependent and ouabain-sensitive ATPase activities was observed (Fig. 12). In the case of ouabain-insensitive ATPase, the effects of nanocolloids differed: under the effect of TiO_2_, the activity indices were at the control level, while under the effect of ZnO, they increased to 121.6% (*p* < 0.01, n = 7). A comparison of the results of studies of membrane ATPase activities under burdening of rats with ZnO and TiO_2_ nanocolloids for 6 months and 30 days^2^ revealed different effects in terms of direction and magnitude. Apparently, due to adaptation processes, the activities of these enzymes undergo the smallest changes under the chronic long-term action of nanocolloids. Thus, our studies showed that chronic exposure to ZnO and TiO_2_ ANs significantly increases the activity of transaminases in the blood plasma and inhibits lipid metabolism, which indicates the impairment of liver function. In addition, under the chronic in vivo effect of the ANs, we observed an increase in the activity of Na^+^,К^+^-ATPase of the plasma membrane, which can cause changes in the functioning of cells.

## Materials and methods

In the in vivo experiments, 8-week-old Wistar rats of both genders were used. The rats were kept in standard vivarium conditions (at room temperature of 20 ± 2 °C, relative humidity of 50–70%, and light-darkness cycle of 12:12 h). The intragastric dose of aqueous ZnO and TiO_2_ nanocolloids (in terms of 3 mg/kg/day of dry matter) was selected under the protocols^46,47^ regarding the probable daily limits of this nanomaterial, which can cause toxic manifestations, > 50 mg/person/day or 0.7 mg/kg/day, as calculated per weight unit. The bodyweight of rats was checked every 4–6 days. The rats of the experimental groups were intragastrically burdened with nanocolloids for six months.

The experiments with animals were conducted under the guidelines of the European Convention for the Protection of Vertebrate Animals used for Experimental and Other Scientific Purposes (Strasbourg, 1986). The experimental protocols were approved by the Commission on bioethics at Educational and Scientific Center "Institute of Biology and Medicine" of Taras Shevchenko National University of Kyiv (protocol № 3 of May 2, 2019). All methods are reported in accordance with ARRIVE guidelines (https://arriveguidelines.org). The animals were euthanized by cervical dislocation under light ether anesthesia.

The in vitro experiments were performed on isolated preparations of circular SMs of gastric *antrum piloricum* and SMs of *caecum* of the large intestine of rats. The tenzometric method in the isometric mode was used to register the spontaneous contractile activity of SMs with the subsequent calculations according to the method^48^ of mechanokinetic parameters of SM contractions and AU contraction indices. Acetylcholine-induced normalized maximal velocities of the GIT SM contraction-relaxation cycles were calculated according to^49^.

The normal Krebs solution with the following concentration of constituents (in mM) was used in the experiments: NaCl—120.4, KCl—5.9, NaHCO_3_—15.5, NaH_2_PO_4_—1.2, MgCl_2_—1.2, CaCl_2_—2.5, glucose—11.5, pH 7.4. The high-potassium Krebs solution with the concentration of K^+^ ions (80 mM) was prepared by replacing the required amount of Na^+^ ions in the normal Krebs solution with the equimolar amount of K^+^ ions. Acetylcholine (AC), (Sigma, USA) was used in the concentration of 10^−5^ M.

### Nanoparticle powders

Commercial nanomaterials: ultradispersed ZnO and TiO_**2**_ powders (PlasmaChem GmbH, Berlin, Germany) were used in the work. Analysis of the NPs’ X-ray powder diffraction (XRPD) patterns obtained by us^2^ using a Shimadzu XRPD-6000 diffractometer (Cu Kα radiation; λ = 1.5405 Å) showed that TiO_**2**_ NP powder consists of anatase and rutile crystallites, while ZnO NPs have wurtzite structure (JCPDS card number 36-1451). ZnO NPs were mostly spherical, while TiO_**2**_ NPs were rectangular in shape. Using transmission electron microscopy (TEM)/high-resolution TEM (HRTM), we determined that the size of TiO_**2**_ NPs is (25 ± 5) nm; the size of ZnO NPs ranges from 17 to 70 nm with a significant predominance of NPs with an average size of about 25 nm. ZnO and TiO_**2**_ NPs had a pronounced crystallinity.

### Aqueous nanocolloids

To obtain aqueous nanocolloids, ultradispersed powders of ZnO and TiO_2_ NPs in the above concentration were dispersed in water and subjected to sonication (28 kHz) for 10 min, incubated for 8 h, and then sonicated again, immediately before the beginning of the experiments. Aggregation resistance of nanocolloids was assessed by measuring the ζ-potential using the Zetasizer Nano (Malvern Panalytical) device. The measurement results showed that ζ-potentials of ZnO and TiO_2_ aqueous nanocolloids and are (+ 28) mV and (+ 32) mV, respectively. According to (TEM)/HTEM and related methods, we found^2^ that the average size of TiO_2_ NPs in the aqueous medium decreased compared to the dry phase. At the same time, no noticeable changes in the crystallinity for the anatase and rutile phases were observed. When introduced into water, the average size of ZnO NPs decreased and was of the same order as that of TiO_2_ NPs. The crystallinity of the core of the core–shell structure was preserved.

#### Biochemical markers

The rat blood plasma was obtained by centrifugation of whole blood and immediately used to determine biochemical markers of the functional state of the liver (the activity of alanine aminotransferase and aspartate aminotransferase, bilirubin concentration, and the thymol test) and lipid concentrations^2^. ALT and AST activities were determined by Reitman-Fresnel method, total and direct bilirubin were determined by Endraschik method and thymol test was performed using thymol reagent checking the test kits (R&D enterprise Felicity-Diagnostics, Ukraine).

We determined the concentration of the following compounds in the blood plasma (mg%): phospholipids, cholesterol (CHOL), cholesterol esters (ECHOL), free fatty acids, triglycerides. Lipids were divided by the method of thin-layer chromatography^49^. Chromatographic separation of lipid components of plasma was carried out on “Silufol” plates. After treatment with an aqueous solution of phosphomolybdic acid, a quantitative assessment of the color intensity of each fraction was performed using a densitometer DO-1 M (“Shimadzu”, Japan, λ 620 nm)^50^.

Blood cell mass was used to obtain erythrocyte plasma membrane preparations by the slightly modified Dodge’s method. Plasma membrane preparations were used to determine the ATPase activities of the primary active ion transport systems (total Mg^2+^, Na^+^, K^+^-ATPase, basal Mg^2+^-ATPase and Na^+^, K^+^-ATPase). The protein concentration in the preparations of the erythrocyte plasma membranes (PM) was determined by Lowry’s method^51^. Total Mg^2+^, Na^+^, K^+^-ATPase activity was determined in the fraction of erythrocyte PMs in the standard incubation medium (in mM): 1 ATP, 3 MgCl_2_, 125 NaCl, 25 KCl, 1 EGTA, 20 Hepes-Tris-buffer (pH 7.4), 1 NaN_3_ (inhibitor of mitochondria ATPase), 0.1 µm thapsigargin (the selective inhibitor of Ca^2+^,Mg^2+^-ATPase of endoplasmatic reticulum) and 0.1% digitonin (the factor of PM perforation), at 37 °C. The Mg^2+^-ATPase activity was determined by the presence of a selective inhibitor Na^+^,K^+^-ATPase ouabain (1 mM) in the incubation medium. The Na^+^, K^+^-ATP activity was calculated as the difference between the total Mg^2+^, Na^+^, K^+^-ATPase and the ouabain-insensitive Mg^2+^-ATPase activity^52,53^.

This paper presents a statistical analysis of the experimental data obtained in the study and processed by the variation statistics methods using the Origin Pro 8 software. The samples were checked to belong to normally distributed general populations according to the Shapiro–Wilk criterion. The dispersion analysis was used to determine reliable differences between the mean values of samplings, and the post-test comparison was made using the Tukey test. In all cases, the results were reliable on the condition of the probability value *p* under 5% (*p* < 0.05). The obtained results were presented as the arithmetic mean ± standard error of the mean value, and the *n* value was determined by the total in the number of experiments.

## Conclusion

Our work addressed a number of questions regarding the effects of ZnO and TiO_2_ nanocolloids administered for 6 months to rats on the mechanisms regulating the motility of gastrointestinal tract and hepatobiliary system of these animals. Below are the most noteworthy findings of the study.

Firstly, the fundamental principle of distribution of physiologically relevant differences in numeric values of mechanokinetic parameters and AU indices for spontaneous contractions of smooth muscles regulated by a heterogeneous population of pacemaker cells in different parts of the gastrointestinal tract was established. These dependencies differed in different age groups of rats.

Secondly, violations of the above-mentioned principles of maintaining different numeric values of mechanokinetic parameters and AU indices of spontaneous contractions of circular SMs of the gastric *antrum* and *caecum* of the large intestine were detected under chronic intragastrical burdening of rats with ZnO and TiO_2_ ANs for 6 months. A comparison of the obtained results with the results of our previous study, whereby rats were exposed to ZnO and TiO_2_ ANs for 30 days, revealed that the greatest changes occur during the short-term action of ZnO and the chronic action of TiO_2_. The general principle of action of these nanocolloids consists in modulation, inversion, and bringing the numeric values of mechanokinetic parameters of spontaneous contractions of SMs in different parts of the gastrointestinal tract to the same level, which can cause its pathological changes.

According to our results, there were changes in the M3- and M2-dependent mechanisms of regulation of cholinergic excitation of both the *antrum* and the *caecum* SMs under intragastrical burdening of rats with ZnO and TiO_2_ ANs. In terms of the magnitude of the changes, these effects were more pronounced under a short-term exposure of rats to these nanocolloids.

The molecular docking of ZnO and TiO_2_ NPs with *myosin II* molecules showed that these nanoparticles do not compete with actin for binding sites with this protein molecule, however they form bonds with a number of its amino acids, which are vital for conformational transformations of this molecule during smooth muscle contraction.

The study also revealed that chronic exposure to ZnO and TiO_2_ ANs is accompanied by an increase in total Mg^2+^-dependent, ouabain-sensitive and ouabain-insensitive (ZnO) ATPase activities of erythrocyte plasma membranes, which indicates that the systems of primary active ion transport of the plasma membrane adapt to the chronic action of these nanocolloids. The above-mentioned changes were significantly smaller than the corresponding changes in rats exposed to ZnO and TiO_2_ ANs for a short period of time. In addition, the studies of rat blood plasma preparations showed that under the same conditions there is a significant increase in the activity of marker liver enzymes—alanine aminotransferase and aspartate aminotransferase, and disruption of the lipid profile, which indicates the hepatotoxic effect of these nanocolloids.

## Data Availability

The datasets obtained during and/or analyzed during the current study are available from the corresponding author on reasonable request.

## Abbreviations

AC: Acetylcholine
ACE: Atomic contact energy
ALT: Alanine aminotransferase
AN: Aqueous nanocolloid
AST: Aspartate aminotransferase
AU: Alexandria units
CHOL: Cholesterol
ECHOL: Cholesterol esters
GIT: Gastrointestinal tract
HFA: Higher fatty acids
ICC: Interstitial cells of Cajal
NPs: Nanoparticles
PM: Plasma membranes
SMs: Smooth muscles
SMC: Smooth muscle cells
VGC: Voltage-gated ion channels
